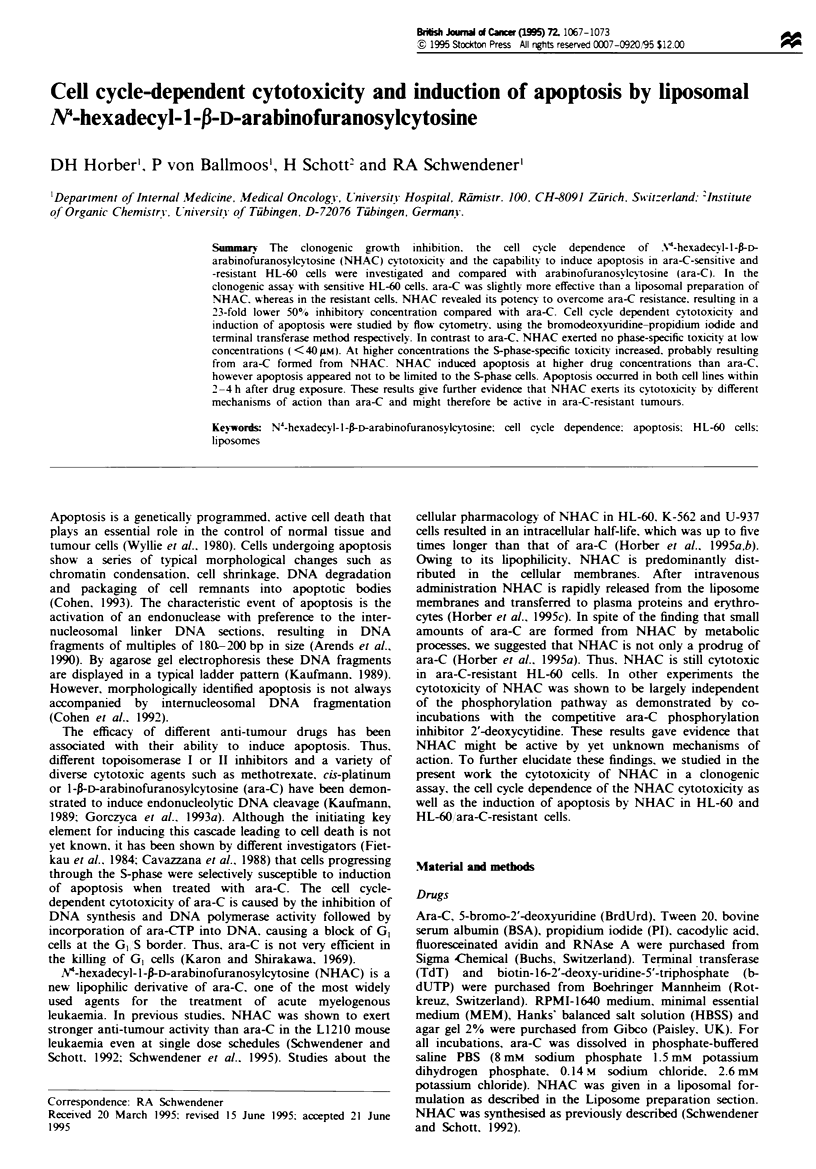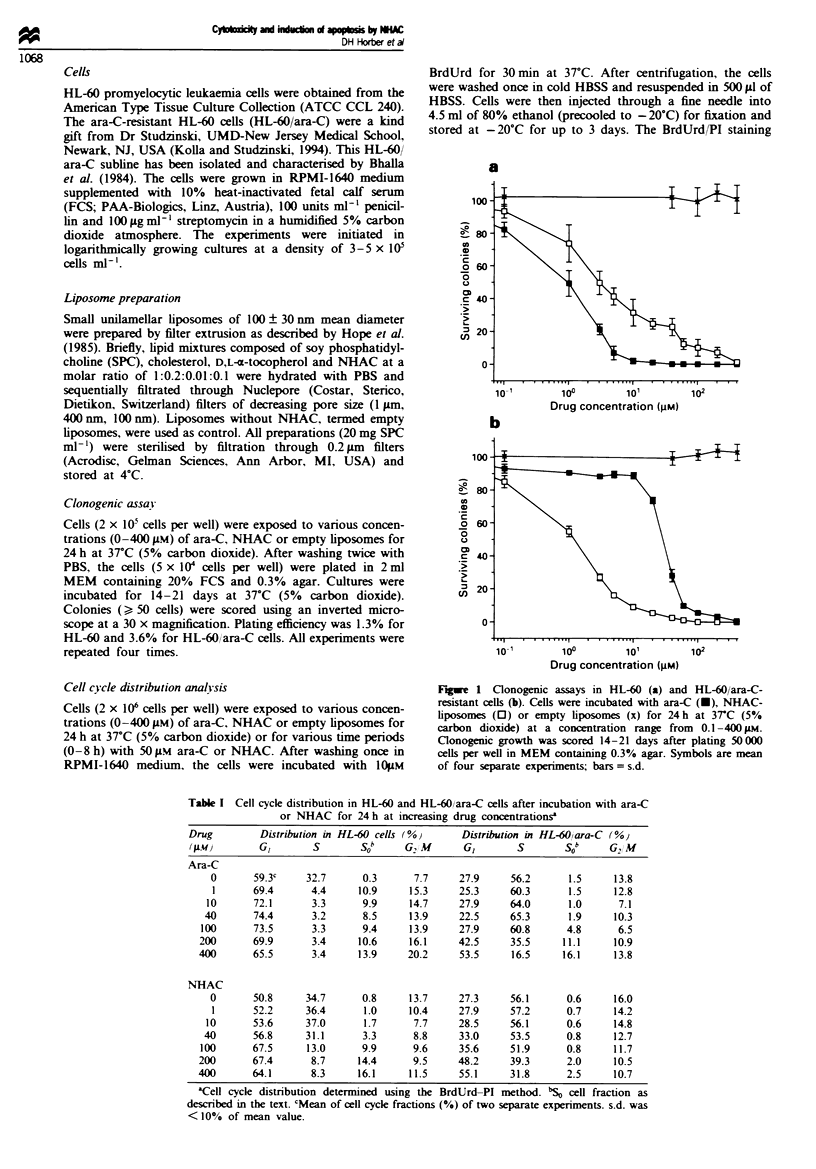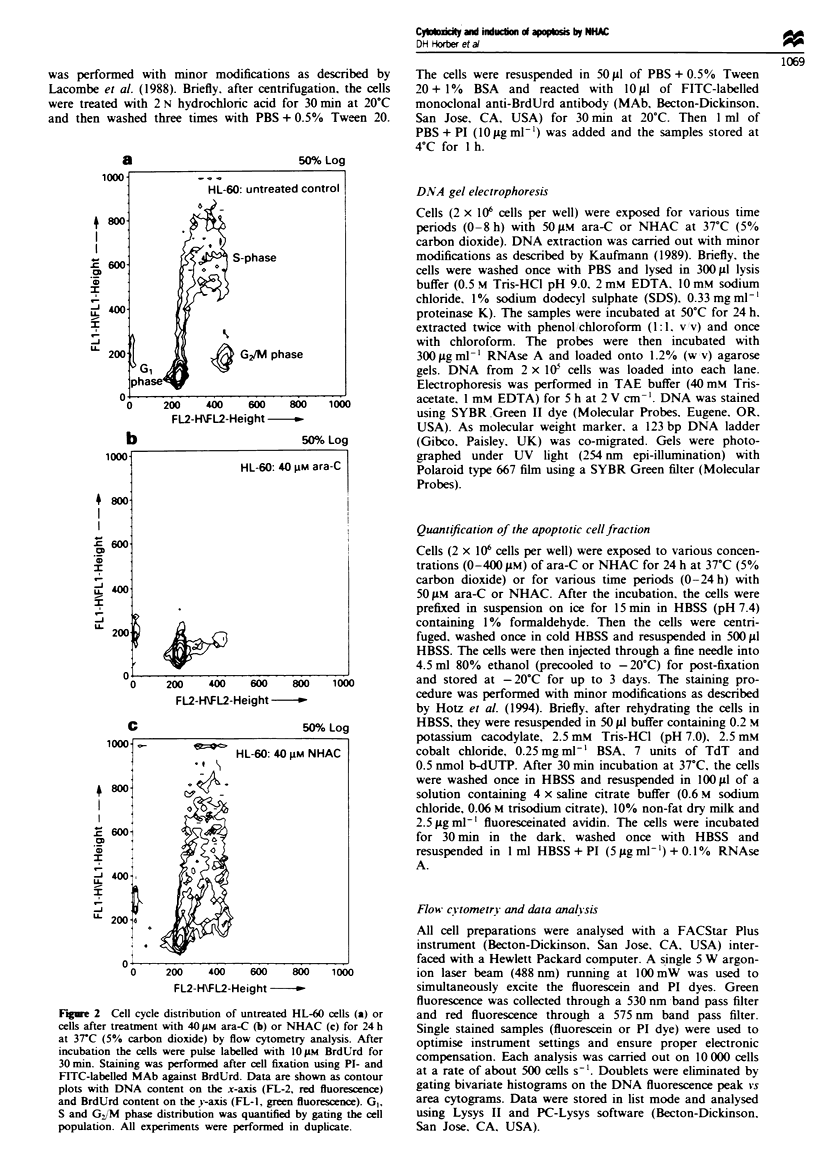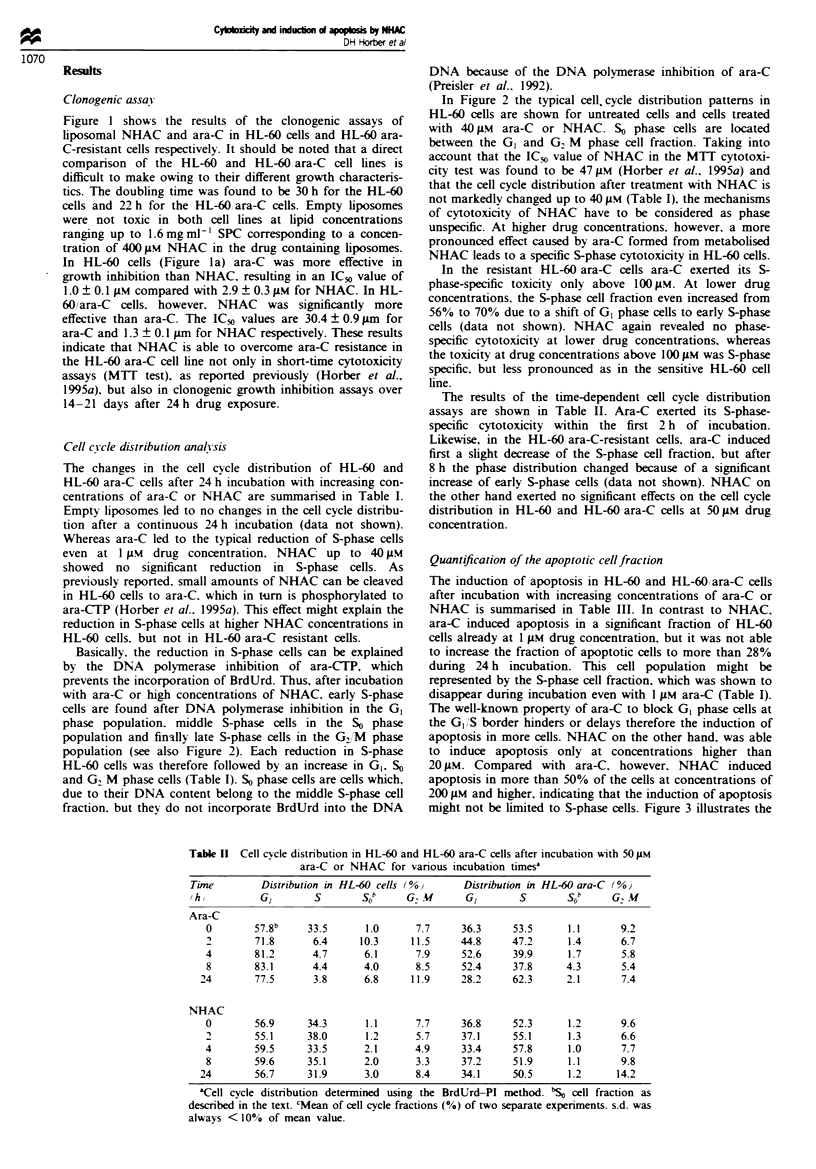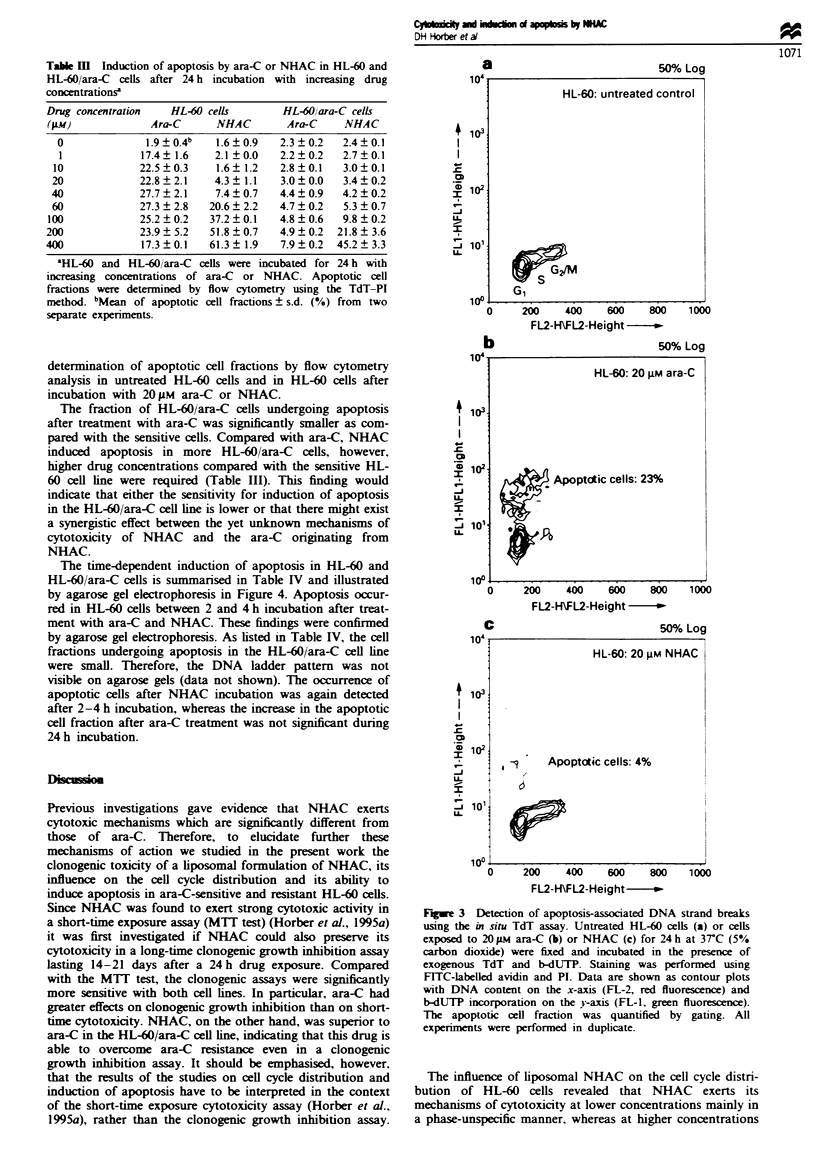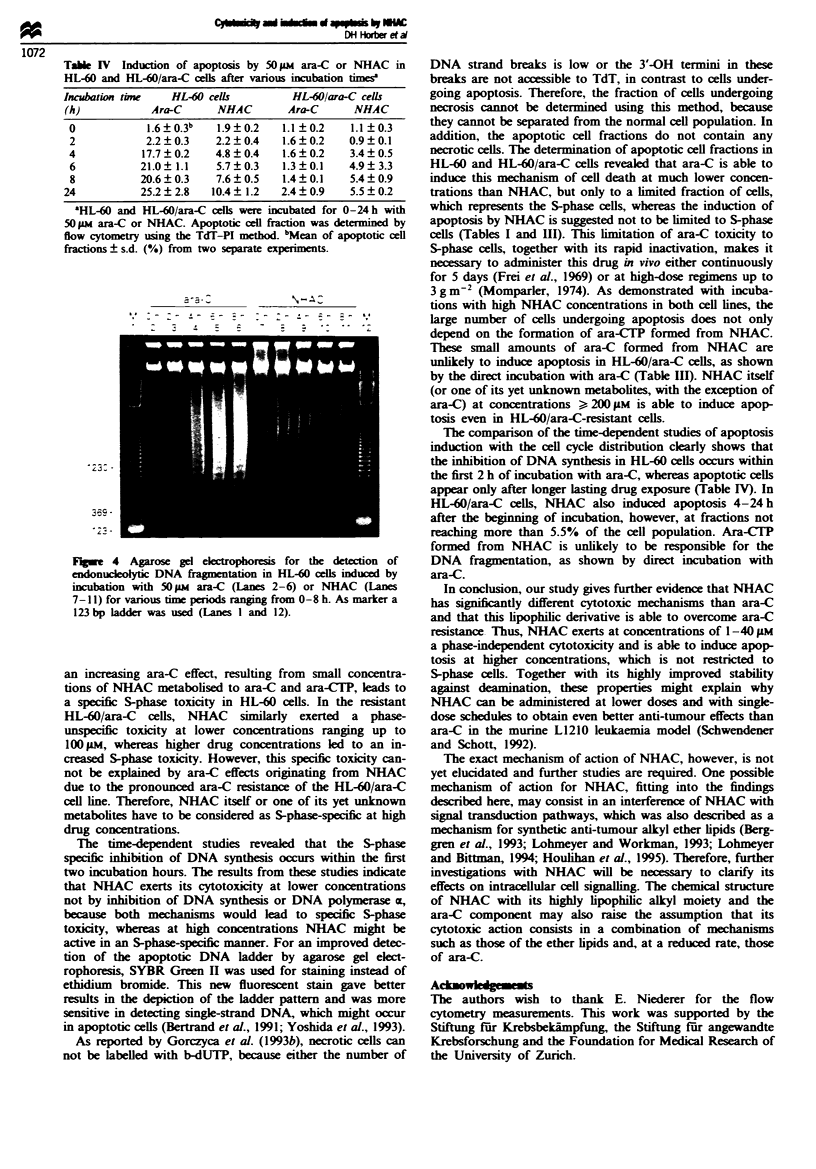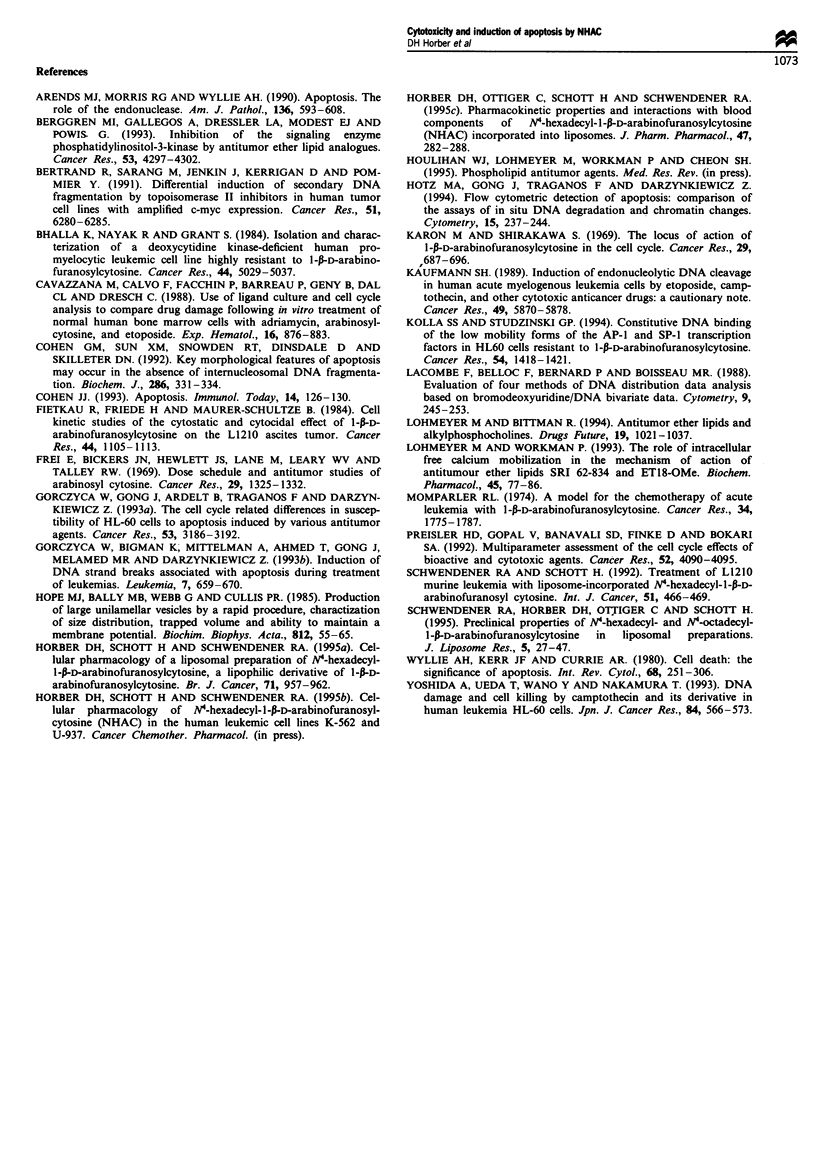# Cell cycle-dependent cytotoxicity and induction of apoptosis by liposomal N4-hexadecyl-1-beta-D-arabinofuranosylcytosine.

**DOI:** 10.1038/bjc.1995.466

**Published:** 1995-11

**Authors:** D. H. Horber, P. von Ballmoos, H. Schott, R. A. Schwendener

**Affiliations:** Department of Internal Medicine, Medical Oncology, University Hospital, Zürich, Switzerland.

## Abstract

**Images:**


British Journal of Cancer (1995) 72, 1067-1073

© 1995 Stockton Press All rights reserved 0007-0920/95 $12.00

### Cell cycle-dependent cytotoxicity and induction of apoptosis by liposomal $N^4$-hexadecyl-1-β-D-arabinofuranosylcytosine

DH Horber[1], P von Ballmoos[1], H Schott[2] and RA Schwendener[1]

[1]*Department of Internal Medicine, Medical Oncology, University Hospital, Rämistr. 100, CH-8091 Zürich, Switzerland;* [2]*Institute of Organic Chemistry, University of Tübingen, D-72076 Tübingen, Germany.*

**Summary** The clonogenic growth inhibition, the cell cycle dependence of $N^4$-hexadecyl-1-β-D-arabinofuranosylcytosine (NHAC) cytotoxicity and the capability to induce apoptosis in ara-C-sensitive and -resistant HL-60 cells were investigated and compared with arabinofuranosylcytosine (ara-C). In the clonogenic assay with sensitive HL-60 cells, ara-C was slightly more effective than a liposomal preparation of NHAC, whereas in the resistant cells, NHAC revealed its potency to overcome ara-C resistance, resulting in a 23-fold lower 50% inhibitory concentration compared with ara-C. Cell cycle dependent cytotoxicity and induction of apoptosis were studied by flow cytometry, using the bromodeoxyuridine–propidium iodide and terminal transferase method respectively. In contrast to ara-C, NHAC exerted no phase-specific toxicity at low concentrations (<40 μM). At higher concentrations the S-phase-specific toxicity increased, probably resulting from ara-C formed from NHAC. NHAC induced apoptosis at higher drug concentrations than ara-C, however apoptosis appeared not to be limited to the S-phase cells. Apoptosis occurred in both cell lines within 2–4 h after drug exposure. These results give further evidence that NHAC exerts its cytotoxicity by different mechanisms of action than ara-C and might therefore be active in ara-C-resistant tumours.

**Keywords:** $N^4$-hexadecyl-1-β-D-arabinofuranosylcytosine; cell cycle dependence; apoptosis; HL-60 cells; liposomes

Apoptosis is a genetically programmed, active cell death that plays an essential role in the control of normal tissue and tumour cells (Wyllie *et al.*, 1980). Cells undergoing apoptosis show a series of typical morphological changes such as chromatin condensation, cell shrinkage, DNA degradation and packaging of cell remnants into apoptotic bodies (Cohen, 1993). The characteristic event of apoptosis is the activation of an endonuclease with preference to the internucleosomal linker DNA sections, resulting in DNA fragments of multiples of 180–200 bp in size (Arends *et al.*, 1990). By agarose gel electrophoresis these DNA fragments are displayed in a typical ladder pattern (Kaufmann, 1989). However, morphologically identified apoptosis is not always accompanied by internucleosomal DNA fragmentation (Cohen *et al.*, 1992).

The efficacy of different anti-tumour drugs has been associated with their ability to induce apoptosis. Thus, different topoisomerase I or II inhibitors and a variety of diverse cytotoxic agents such as methotrexate, *cis*-platinum or 1-β-D-arabinofuranosylcytosine (ara-C) have been demonstrated to induce endonucleolytic DNA cleavage (Kaufmann, 1989; Gorczyca *et al.*, 1993*a*). Although the initiating key element for inducing this cascade leading to cell death is not yet known, it has been shown by different investigators (Fietkau *et al.*, 1984; Cavazzana *et al.*, 1988) that cells progressing through the S-phase were selectively susceptible to induction of apoptosis when treated with ara-C. The cell cycle-dependent cytotoxicity of ara-C is caused by the inhibition of DNA synthesis and DNA polymerase activity followed by incorporation of ara-CTP into DNA, causing a block of $G_1$ cells at the $G_1$/S border. Thus, ara-C is not very efficient in the killing of $G_1$ cells (Karon and Shirakawa, 1969).

$N^4$-hexadecyl-1-β-D-arabinofuranosylcytosine (NHAC) is a new lipophilic derivative of ara-C, one of the most widely used agents for the treatment of acute myelogenous leukaemia. In previous studies, NHAC was shown to exert stronger anti-tumour activity than ara-C in the L1210 mouse leukaemia even at single dose schedules (Schwendener and Schott, 1992; Schwendener *et al.*, 1995). Studies about the cellular pharmacology of NHAC in HL-60, K-562 and U-937 cells resulted in an intracellular half-life, which was up to five times longer than that of ara-C (Horber *et al.*, 1995*a,b*). Owing to its lipophilicity, NHAC is predominantly distributed in the cellular membranes. After intravenous administration NHAC is rapidly released from the liposome membranes and transferred to plasma proteins and erythrocytes (Horber *et al.*, 1995*c*). In spite of the finding that small amounts of ara-C are formed from NHAC by metabolic processes, we suggested that NHAC is not only a prodrug of ara-C (Horber *et al.*, 1995*a*). Thus, NHAC is still cytotoxic in ara-C-resistant HL-60 cells. In other experiments the cytotoxicity of NHAC was shown to be largely independent of the phosphorylation pathway as demonstrated by co-incubations with the competitive ara-C phosphorylation inhibitor 2′-deoxycytidine. These results gave evidence that NHAC might be active by yet unknown mechanisms of action. To further elucidate these findings, we studied in the present work the cytotoxicity of NHAC in a clonogenic assay, the cell cycle dependence of the NHAC cytotoxicity as well as the induction of apoptosis by NHAC in HL-60 and HL-60/ara-C-resistant cells.

### Material and methods

### *Drugs*

Ara-C, 5-bromo-2′-deoxyuridine (BrdUrd), Tween 20, bovine serum albumin (BSA), propidium iodide (PI), cacodylic acid, fluoresceinated avidin and RNAse A were purchased from Sigma Chemical (Buchs, Switzerland). Terminal transferase (TdT) and biotin-16-2′-deoxy-uridine-5′-triphosphate (b-dUTP) were purchased from Boehringer Mannheim (Rotkreuz, Switzerland). RPMI-1640 medium, minimal essential medium (MEM), Hanks' balanced salt solution (HBSS) and agar gel 2% were purchased from Gibco (Paisley, UK). For all incubations, ara-C was dissolved in phosphate-buffered saline PBS (8 mM sodium phosphate 1.5 mM potassium dihydrogen phosphate, 0.14 M sodium chloride, 2.6 mM potassium chloride). NHAC was given in a liposomal formulation as described in the Liposome preparation section. NHAC was synthesised as previously described (Schwendener and Schott, 1992).

Correspondence: RA Schwendener
Received 20 March 1995; revised 15 June 1995; accepted 21 June 1995

### Cells

HL-60 promyelocytic leukaemia cells were obtained from the American Type Tissue Culture Collection (ATCC CCL 240). The ara-C-resistant HL-60 cells (HL-60/ara-C) were a kind gift from Dr Studzinski, UMD-New Jersey Medical School, Newark, NJ, USA (Kolla and Studzinski, 1994). This HL-60/ara-C subline has been isolated and characterised by Bhalla *et al.* (1984). The cells were grown in RPMI-1640 medium supplemented with 10% heat-inactivated fetal calf serum (FCS; PAA-Biologics, Linz, Austria), 100 units $ml^{-1}$ penicillin and 100 $\mu g\ ml^{-1}$ streptomycin in a humidified 5% carbon dioxide atmosphere. The experiments were initiated in logarithmically growing cultures at a density of $3-5 \times 10^5$ cells $ml^{-1}$.

### Liposome preparation

Small unilamellar liposomes of $100 \pm 30$ nm mean diameter were prepared by filter extrusion as described by Hope *et al.* (1985). Briefly, lipid mixtures composed of soy phosphatidylcholine (SPC), cholesterol, D,L-α-tocopherol and NHAC at a molar ratio of 1:0.2:0.01:0.1 were hydrated with PBS and sequentially filtrated through Nuclepore (Costar, Sterico, Dietikon, Switzerland) filters of decreasing pore size (1 μm, 400 nm, 100 nm). Liposomes without NHAC, termed empty liposomes, were used as control. All preparations (20 mg SPC $ml^{-1}$) were sterilised by filtration through 0.2 μm filters (Acrodisc, Gelman Sciences, Ann Arbor, MI, USA) and stored at 4°C.

### Clonogenic assay

Cells ($2 \times 10^5$ cells per well) were exposed to various concentrations (0–400 μM) of ara-C, NHAC or empty liposomes for 24 h at 37°C (5% carbon dioxide). After washing twice with PBS, the cells ($5 \times 10^4$ cells per well) were plated in 2 ml MEM containing 20% FCS and 0.3% agar. Cultures were incubated for 14–21 days at 37°C (5% carbon dioxide). Colonies (≥ 50 cells) were scored using an inverted microscope at a 30 × magnification. Plating efficiency was 1.3% for HL-60 and 3.6% for HL-60/ara-C cells. All experiments were repeated four times.

### Cell cycle distribution analysis

Cells ($2 \times 10^6$ cells per well) were exposed to various concentrations (0–400 μM) of ara-C, NHAC or empty liposomes for 24 h at 37°C (5% carbon dioxide) or for various time periods (0–8 h) with 50 μM ara-C or NHAC. After washing once in RPMI-1640 medium, the cells were incubated with 10μM BrdUrd for 30 min at 37°C. After centrifugation, the cells were washed once in cold HBSS and resuspended in 500 μl of HBSS. Cells were then injected through a fine needle into 4.5 ml of 80% ethanol (precooled to −20°C) for fixation and stored at −20°C for up to 3 days. The BrdUrd/PI staining

**Figure 1** Clonogenic assays in HL-60 (**a**) and HL-60/ara-C-resistant cells (**b**). Cells were incubated with ara-C (■), NHAC-liposomes (□) or empty liposomes (x) for 24 h at 37°C (5% carbon dioxide) at a concentration range from 0.1–400 μM. Clonogenic growth was scored 14–21 days after plating 50 000 cells per well in MEM containing 0.3% agar. Symbols are mean of four separate experiments; bars = s.d.

**Table I** Cell cycle distribution in HL-60 and HL-60/ara-C cells after incubation with ara-C or NHAC for 24 h at increasing drug concentrations[a]

| *Drug (μM)* | *Distribution in HL-60 cells (%)* $G_1$ | $S$ | $S_0^b$ | $G_2/M$ | *Distribution in HL-60/ara-C (%)* $G_1$ | $S$ | $S_0^b$ | $G_2/M$ |
|---|---|---|---|---|---|---|---|---|
| Ara-C | | | | | | | | |
| 0 | 59.3[c] | 32.7 | 0.3 | 7.7 | 27.9 | 56.2 | 1.5 | 13.8 |
| 1 | 69.4 | 4.4 | 10.9 | 15.3 | 25.3 | 60.3 | 1.5 | 12.8 |
| 10 | 72.1 | 3.3 | 9.9 | 14.7 | 27.9 | 64.0 | 1.0 | 7.1 |
| 40 | 74.4 | 3.2 | 8.5 | 13.9 | 22.5 | 65.3 | 1.9 | 10.3 |
| 100 | 73.5 | 3.3 | 9.4 | 13.9 | 27.9 | 60.8 | 4.8 | 6.5 |
| 200 | 69.9 | 3.4 | 10.6 | 16.1 | 42.5 | 35.5 | 11.1 | 10.9 |
| 400 | 65.5 | 3.4 | 13.9 | 20.2 | 53.5 | 16.5 | 16.1 | 13.8 |
| NHAC | | | | | | | | |
| 0 | 50.8 | 34.7 | 0.8 | 13.7 | 27.3 | 56.1 | 0.6 | 16.0 |
| 1 | 52.2 | 36.4 | 1.0 | 10.4 | 27.9 | 57.2 | 0.7 | 14.2 |
| 10 | 53.6 | 37.0 | 1.7 | 7.7 | 28.5 | 56.1 | 0.6 | 14.8 |
| 40 | 56.8 | 31.1 | 3.3 | 8.8 | 33.0 | 53.5 | 0.8 | 12.7 |
| 100 | 67.5 | 13.0 | 9.9 | 9.6 | 35.6 | 51.9 | 0.8 | 11.7 |
| 200 | 67.4 | 8.7 | 14.4 | 9.5 | 48.2 | 39.3 | 2.0 | 10.5 |
| 400 | 64.1 | 8.3 | 16.1 | 11.5 | 55.1 | 31.8 | 2.5 | 10.7 |

[a]Cell cycle distribution determined using the BrdUrd–PI method. [b]$S_0$ cell fraction as described in the text. [c]Mean of cell cycle fractions (%) of two separate experiments. s.d. was < 10% of mean value.

was performed with minor modifications as described by Lacombe *et al.* (1988). Briefly, after centrifugation, the cells were treated with 2 N hydrochloric acid for 30 min at 20°C and then washed three times with PBS + 0.5% Tween 20.

**Figure 2** Cell cycle distribution of untreated HL-60 cells (**a**) or cells after treatment with 40 μM ara-C (**b**) or NHAC (**c**) for 24 h at 37°C (5% carbon dioxide) by flow cytometry analysis. After incubation the cells were pulse labelled with 10 μM BrdUrd for 30 min. Staining was performed after cell fixation using PI- and FITC-labelled MAb against BrdUrd. Data are shown as contour plots with DNA content on the *x*-axis (FL-2, red fluorescence) and BrdUrd content on the *y*-axis (FL-1, green fluorescence). $G_1$, S and $G_2$/M phase distribution was quantified by gating the cell population. All experiments were performed in duplicate.

The cells were resuspended in 50 μl of PBS + 0.5% Tween 20 + 1% BSA and reacted with 10 μl of FITC-labelled monoclonal anti-BrdUrd antibody (MAb, Becton-Dickinson, San Jose, CA, USA) for 30 min at 20°C. Then 1 ml of PBS + PI (10 μg $ml^{-1}$) was added and the samples stored at 4°C for 1 h.

### *DNA gel electrophoresis*

Cells (2 × $10^6$ cells per well) were exposed for various time periods (0–8 h) with 50 μM ara-C or NHAC at 37°C (5% carbon dioxide). DNA extraction was carried out with minor modifications as described by Kaufmann (1989). Briefly, the cells were washed once with PBS and lysed in 300 μl lysis buffer (0.5 M Tris-HCl pH 9.0, 2 mM EDTA, 10 mM sodium chloride, 1% sodium dodecyl sulphate (SDS), 0.33 mg $ml^{-1}$ proteinase K). The samples were incubated at 50°C for 24 h, extracted twice with phenol/chloroform (1:1, v/v) and once with chloroform. The probes were then incubated with 300 μg $ml^{-1}$ RNAse A and loaded onto 1.2% (w/v) agarose gels. DNA from 2 × $10^5$ cells was loaded into each lane. Electrophoresis was performed in TAE buffer (40 mM Tris-acetate, 1 mM EDTA) for 5 h at 2 V $cm^{-1}$. DNA was stained using SYBR Green II dye (Molecular Probes, Eugene, OR, USA). As molecular weight marker, a 123 bp DNA ladder (Gibco, Paisley, UK) was co-migrated. Gels were photographed under UV light (254 nm epi-illumination) with Polaroid type 667 film using a SYBR Green filter (Molecular Probes).

### *Quantification of the apoptotic cell fraction*

Cells (2 × $10^6$ cells per well) were exposed to various concentrations (0–400 μM) of ara-C or NHAC for 24 h at 37°C (5% carbon dioxide) or for various time periods (0–24 h) with 50 μM ara-C or NHAC. After the incubation, the cells were prefixed in suspension on ice for 15 min in HBSS (pH 7.4) containing 1% formaldehyde. Then the cells were centrifuged, washed once in cold HBSS and resuspended in 500 μl HBSS. The cells were then injected through a fine needle into 4.5 ml 80% ethanol (precooled to −20°C) for post-fixation and stored at −20°C for up to 3 days. The staining procedure was performed with minor modifications as described by Hotz *et al.* (1994). Briefly, after rehydrating the cells in HBSS, they were resuspended in 50 μl buffer containing 0.2 M potassium cacodylate, 2.5 mM Tris-HCl (pH 7.0), 2.5 mM cobalt chloride, 0.25 mg $ml^{-1}$ BSA, 7 units of TdT and 0.5 nmol b-dUTP. After 30 min incubation at 37°C, the cells were washed once in HBSS and resuspended in 100 μl of a solution containing 4 × saline citrate buffer (0.6 M sodium chloride, 0.06 M trisodium citrate), 10% non-fat dry milk and 2.5 μg $ml^{-1}$ fluoresceinated avidin. The cells were incubated for 30 min in the dark, washed once with HBSS and resuspended in 1 ml HBSS + PI (5 μg $ml^{-1}$) + 0.1% RNAse A.

### *Flow cytometry and data analysis*

All cell preparations were analysed with a FACStar Plus instrument (Becton-Dickinson, San Jose, CA, USA) interfaced with a Hewlett Packard computer. A single 5 W argon-ion laser beam (488 nm) running at 100 mW was used to simultaneously excite the fluorescein and PI dyes. Green fluorescence was collected through a 530 nm band pass filter and red fluorescence through a 575 nm band pass filter. Single stained samples (fluorescein or PI dye) were used to optimise instrument settings and ensure proper electronic compensation. Each analysis was carried out on 10 000 cells at a rate of about 500 cells $s^{-1}$. Doublets were eliminated by gating bivariate histograms on the DNA fluorescence peak *vs* area cytograms. Data were stored in list mode and analysed using Lysys II and PC-Lysys software (Becton-Dickinson, San Jose, CA, USA).

### Results

### Clonogenic assay

Figure 1 shows the results of the clonogenic assays of liposomal NHAC and ara-C in HL-60 cells and HL-60/ara-C-resistant cells respectively. It should be noted that a direct comparison of the HL-60 and HL-60/ara-C cell lines is difficult to make owing to their different growth characteristics. The doubling time was found to be 30 h for the HL-60 cells and 22 h for the HL-60/ara-C cells. Empty liposomes were not toxic in both cell lines at lipid concentrations ranging up to 1.6 mg ml$^{-1}$ SPC corresponding to a concentration of 400 μM NHAC in the drug containing liposomes. In HL-60 cells (Figure 1a) ara-C was more effective in growth inhibition than NHAC, resulting in an $IC_{50}$ value of $1.0 \pm 0.1$ μM compared with $2.9 \pm 0.3$ μM for NHAC. In HL-60/ara-C cells, however, NHAC was significantly more effective than ara-C. The $IC_{50}$ values are $30.4 \pm 0.9$ μm for ara-C and $1.3 \pm 0.1$ μm for NHAC respectively. These results indicate that NHAC is able to overcome ara-C resistance in the HL-60/ara-C cell line not only in short-time cytotoxicity assays (MTT test), as reported previously (Horber *et al*., 1995*a*), but also in clonogenic growth inhibition assays over 14–21 days after 24 h drug exposure.

### Cell cycle distribution analysis

The changes in the cell cycle distribution of HL-60 and HL-60 ara-C cells after 24 h incubation with increasing concentrations of ara-C or NHAC are summarised in Table I. Empty liposomes led to no changes in the cell cycle distribution after a continuous 24 h incubation (data not shown). Whereas ara-C led to the typical reduction of S-phase cells even at 1 μM drug concentration, NHAC up to 40 μM showed no significant reduction in S-phase cells. As previously reported, small amounts of NHAC can be cleaved in HL-60 cells to ara-C, which in turn is phosphorylated to ara-CTP (Horber *et al*., 1995*a*). This effect might explain the reduction in S-phase cells at higher NHAC concentrations in HL-60 cells, but not in HL-60/ara-C resistant cells.

Basically, the reduction in S-phase cells can be explained by the DNA polymerase inhibition of ara-CTP, which prevents the incorporation of BrdUrd. Thus, after incubation with ara-C or high concentrations of NHAC, early S-phase cells are found after DNA polymerase inhibition in the $G_1$ phase population, middle S-phase cells in the $S_0$ phase population and finally late S-phase cells in the $G_2/M$ phase population (see also Figure 2). Each reduction in S-phase HL-60 cells was therefore followed by an increase in $G_1$, $S_0$ and $G_2/M$ phase cells (Table I). $S_0$ phase cells are cells which, due to their DNA content belong to the middle S-phase cell fraction, but they do not incorporate BrdUrd into the DNA DNA because of the DNA polymerase inhibition of ara-C (Preisler *et al*., 1992).

In Figure 2 the typical cell cycle distribution patterns in HL-60 cells are shown for untreated cells and cells treated with 40 μM ara-C or NHAC. $S_0$ phase cells are located between the $G_1$ and $G_2/M$ phase cell fraction. Taking into account that the $IC_{50}$ value of NHAC in the MTT cytotoxicity test was found to be 47 μM (Horber *et al*., 1995*a*) and that the cell cycle distribution after treatment with NHAC is not markedly changed up to 40 μM (Table I), the mechanisms of cytotoxicity of NHAC have to be considered as phase unspecific. At higher drug concentrations, however, a more pronounced effect caused by ara-C formed from metabolised NHAC leads to a specific S-phase cytotoxicity in HL-60 cells.

In the resistant HL-60/ara-C cells ara-C exerted its S-phase-specific toxicity only above 100 μM. At lower drug concentrations, the S-phase cell fraction even increased from 56% to 70% due to a shift of $G_1$ phase cells to early S-phase cells (data not shown). NHAC again revealed no phase-specific cytotoxicity at lower drug concentrations, whereas the toxicity at drug concentrations above 100 μM was S-phase specific, but less pronounced as in the sensitive HL-60 cell line.

The results of the time-dependent cell cycle distribution assays are shown in Table II. Ara-C exerted its S-phase-specific cytotoxicity within the first 2 h of incubation. Likewise, in the HL-60/ara-C-resistant cells, ara-C induced first a slight decrease of the S-phase cell fraction, but after 8 h the phase distribution changed because of a significant increase of early S-phase cells (data not shown). NHAC on the other hand exerted no significant effects on the cell cycle distribution in HL-60 and HL-60/ara-C cells at 50 μM drug concentration.

### Quantification of the apoptotic cell fraction

The induction of apoptosis in HL-60 and HL-60/ara-C cells after incubation with increasing concentrations of ara-C or NHAC is summarised in Table III. In contrast to NHAC, ara-C induced apoptosis in a significant fraction of HL-60 cells already at 1 μM drug concentration, but it was not able to increase the fraction of apoptotic cells to more than 28% during 24 h incubation. This cell population might be represented by the S-phase cell fraction, which was shown to disappear during incubation even with 1 μM ara-C (Table I). The well-known property of ara-C to block $G_1$ phase cells at the $G_1/S$ border hinders or delays therefore the induction of apoptosis in more cells. NHAC on the other hand, was able to induce apoptosis only at concentrations higher than 20 μM. Compared with ara-C, however, NHAC induced apoptosis in more than 50% of the cells at concentrations of 200 μM and higher, indicating that the induction of apoptosis might not be limited to S-phase cells. Figure 3 illustrates the

**Table II** Cell cycle distribution in HL-60 and HL-60 ara-C cells after incubation with 50 μM ara-C or NHAC for various incubation times[a]

| *Time (h)* | *Distribution in HL-60 cells (%)* $G_1$ | S | $S_0^b$ | $G_2/M$ | *Distribution in HL-60 ara-C (%)* $G_1$ | S | $S_0^b$ | $G_2/M$ |
|---|---|---|---|---|---|---|---|---|
| Ara-C | | | | | | | | |
| 0 | 57.8[b] | 33.5 | 1.0 | 7.7 | 36.3 | 53.5 | 1.1 | 9.2 |
| 2 | 71.8 | 6.4 | 10.3 | 11.5 | 44.8 | 47.2 | 1.4 | 6.7 |
| 4 | 81.2 | 4.7 | 6.1 | 7.9 | 52.6 | 39.9 | 1.7 | 5.8 |
| 8 | 83.1 | 4.4 | 4.0 | 8.5 | 52.4 | 37.8 | 4.3 | 5.4 |
| 24 | 77.5 | 3.8 | 6.8 | 11.9 | 28.2 | 62.3 | 2.1 | 7.4 |
| NHAC | | | | | | | | |
| 0 | 56.9 | 34.3 | 1.1 | 7.7 | 36.8 | 52.3 | 1.2 | 9.6 |
| 2 | 55.1 | 38.0 | 1.2 | 5.7 | 37.1 | 55.1 | 1.3 | 6.6 |
| 4 | 59.5 | 33.5 | 2.1 | 4.9 | 33.4 | 57.8 | 1.0 | 7.7 |
| 8 | 59.6 | 35.1 | 2.0 | 3.3 | 37.2 | 51.9 | 1.1 | 9.8 |
| 24 | 56.7 | 31.9 | 3.0 | 8.4 | 34.1 | 50.5 | 1.2 | 14.2 |

[a]Cell cycle distribution determined using the BrdUrd–PI method. [b]$S_0$ cell fraction as described in the text. [c]Mean of cell cycle fractions (%) of two separate experiments, s.d. was always <10% of mean value.

**Table III** Induction of apoptosis by ara-C or NHAC in HL-60 and HL-60/ara-C cells after 24 h incubation with increasing drug concentrations[a]

| *Drug concentration (μM)* | *HL-60 cells Ara-C* | *HL-60 cells NHAC* | *HL-60/ara-C cells Ara-C* | *HL-60/ara-C cells NHAC* |
|---|---|---|---|---|
| 0 | 1.9 ± 0.4[b] | 1.6 ± 0.9 | 2.3 ± 0.2 | 2.4 ± 0.1 |
| 1 | 17.4 ± 1.6 | 2.1 ± 0.0 | 2.2 ± 0.2 | 2.7 ± 0.1 |
| 10 | 22.5 ± 0.3 | 1.6 ± 1.2 | 2.8 ± 0.1 | 3.0 ± 0.1 |
| 20 | 22.8 ± 2.1 | 4.3 ± 1.1 | 3.0 ± 0.0 | 3.4 ± 0.2 |
| 40 | 27.7 ± 2.1 | 7.4 ± 0.7 | 4.4 ± 0.9 | 4.2 ± 0.2 |
| 60 | 27.3 ± 2.8 | 20.6 ± 2.2 | 4.7 ± 0.2 | 5.3 ± 0.7 |
| 100 | 25.2 ± 0.2 | 37.2 ± 0.1 | 4.8 ± 0.6 | 9.8 ± 0.2 |
| 200 | 23.9 ± 5.2 | 51.8 ± 0.7 | 4.9 ± 0.2 | 21.8 ± 3.6 |
| 400 | 17.3 ± 0.1 | 61.3 ± 1.9 | 7.9 ± 0.2 | 45.2 ± 3.3 |

[a]HL-60 and HL-60/ara-C cells were incubated for 24 h with increasing concentrations of ara-C or NHAC. Apoptotic cell fractions were determined by flow cytometry using the TdT–PI method. [b]Mean of apoptotic cell fractions ± s.d. (%) from two separate experiments.

determination of apoptotic cell fractions by flow cytometry analysis in untreated HL-60 cells and in HL-60 cells after incubation with 20 μM ara-C or NHAC.

The fraction of HL-60/ara-C cells undergoing apoptosis after treatment with ara-C was significantly smaller as compared with the sensitive cells. Compared with ara-C, NHAC induced apoptosis in more HL-60/ara-C cells, however, higher drug concentrations compared with the sensitive HL-60 cell line were required (Table III). This finding would indicate that either the sensitivity for induction of apoptosis in the HL-60/ara-C cell line is lower or that there might exist a synergistic effect between the yet unknown mechanisms of cytotoxicity of NHAC and the ara-C originating from NHAC.

The time-dependent induction of apoptosis in HL-60 and HL-60/ara-C cells is summarised in Table IV and illustrated by agarose gel electrophoresis in Figure 4. Apoptosis occurred in HL-60 cells between 2 and 4 h incubation after treatment with ara-C and NHAC. These findings were confirmed by agarose gel electrophoresis. As listed in Table IV, the cell fractions undergoing apoptosis in the HL-60/ara-C cell line were small. Therefore, the DNA ladder pattern was not visible on agarose gels (data not shown). The occurrence of apoptotic cells after NHAC incubation was again detected after 2–4 h incubation, whereas the increase in the apoptotic cell fraction after ara-C treatment was not significant during 24 h incubation.

### Discussion

Previous investigations gave evidence that NHAC exerts cytotoxic mechanisms which are significantly different from those of ara-C. Therefore, to elucidate further these mechanisms of action we studied in the present work the clonogenic toxicity of a liposomal formulation of NHAC, its influence on the cell cycle distribution and its ability to induce apoptosis in ara-C-sensitive and resistant HL-60 cells. Since NHAC was found to exert strong cytotoxic activity in a short-time exposure assay (MTT test) (Horber *et al.*, 1995*a*) it was first investigated if NHAC could also preserve its cytotoxicity in a long-time clonogenic growth inhibition assay lasting 14–21 days after a 24 h drug exposure. Compared with the MTT test, the clonogenic assays were significantly more sensitive with both cell lines. In particular, ara-C had greater effects on clonogenic growth inhibition than on short-time cytotoxicity. NHAC, on the other hand, was superior to ara-C in the HL-60/ara-C cell line, indicating that this drug is able to overcome ara-C resistance even in a clonogenic growth inhibition assay. It should be emphasised, however, that the results of the studies on cell cycle distribution and induction of apoptosis have to be interpreted in the context of the short-time exposure cytotoxicity assay (Horber *et al.*, 1995*a*), rather than the clonogenic growth inhibition assay.

**Figure 3** Detection of apoptosis-associated DNA strand breaks using the *in situ* TdT assay. Untreated HL-60 cells (**a**) or cells exposed to 20 μM ara-C (**b**) or NHAC (**c**) for 24 h at 37°C (5% carbon dioxide) were fixed and incubated in the presence of exogenous TdT and b-dUTP. Staining was performed using FITC-labelled avidin and PI. Data are shown as contour plots with DNA content on the *x*-axis (FL-2, red fluorescence) and b-dUTP incorporation on the *y*-axis (FL-1, green fluorescence). The apoptotic cell fraction was quantified by gating. All experiments were performed in duplicate.

The influence of liposomal NHAC on the cell cycle distribution of HL-60 cells revealed that NHAC exerts its mechanisms of cytotoxicity at lower concentrations mainly in a phase-unspecific manner, whereas at higher concentrations

**Table IV** Induction of apoptosis by 50 μM ara-C or NHAC in HL-60 and HL-60/ara-C cells after various incubation times[a]

| *Incubation time (h)* | *HL-60 cells* | | *HL-60/ara-C cells* | |
|---|---|---|---|---|
| | *Ara-C* | *NHAC* | *Ara-C* | *NHAC* |
| 0 | 1.6 ± 0.3[b] | 1.9 ± 0.2 | 1.1 ± 0.2 | 1.1 ± 0.3 |
| 2 | 2.2 ± 0.3 | 2.2 ± 0.4 | 1.6 ± 0.2 | 0.9 ± 0.1 |
| 4 | 17.7 ± 0.2 | 4.8 ± 0.4 | 1.6 ± 0.2 | 3.4 ± 0.5 |
| 6 | 21.0 ± 1.1 | 5.7 ± 0.3 | 1.3 ± 0.1 | 4.9 ± 3.3 |
| 8 | 20.6 ± 0.3 | 7.6 ± 0.5 | 1.4 ± 0.1 | 5.4 ± 0.9 |
| 24 | 25.2 ± 2.8 | 10.4 ± 1.2 | 2.4 ± 0.9 | 5.5 ± 0.2 |

[a]HL-60 and HL-60/ara-C cells were incubated for 0–24 h with 50 μM ara-C or NHAC. Apoptotic cell fraction was determined by flow cytometry using the TdT-PI method. [b]Mean of apoptotic cell fractions ± s.d. (%) from two separate experiments.

**Figure 4** Agarose gel electrophoresis for the detection of endonucleolytic DNA fragmentation in HL-60 cells induced by incubation with 50 μM ara-C (Lanes 2–6) or NHAC (Lanes 7–11) for various time periods ranging from 0–8 h. As marker a 123 bp ladder was used (Lanes 1 and 12).

an increasing ara-C effect, resulting from small concentrations of NHAC metabolised to ara-C and ara-CTP, leads to a specific S-phase toxicity in HL-60 cells. In the resistant HL-60/ara-C cells, NHAC similarly exerted a phase-unspecific toxicity at lower concentrations ranging up to 100 μM, whereas higher drug concentrations led to an increased S-phase toxicity. However, this specific toxicity cannot be explained by ara-C effects originating from NHAC due to the pronounced ara-C resistance of the HL-60/ara-C cell line. Therefore, NHAC itself or one of its yet unknown metabolites have to be considered as S-phase-specific at high drug concentrations.

The time-dependent studies revealed that the S-phase specific inhibition of DNA synthesis occurs within the first two incubation hours. The results from these studies indicate that NHAC exerts its cytotoxicity at lower concentrations not by inhibition of DNA synthesis or DNA polymerase α, because both mechanisms would lead to specific S-phase toxicity, whereas at high concentrations NHAC might be active in an S-phase-specific manner. For an improved detection of the apoptotic DNA ladder by agarose gel electrophoresis, SYBR Green II was used for staining instead of ethidium bromide. This new fluorescent stain gave better results in the depiction of the ladder pattern and was more sensitive in detecting single-strand DNA, which might occur in apoptotic cells (Bertrand *et al.*, 1991; Yoshida *et al.*, 1993).

As reported by Gorczyca *et al.* (1993*b*), necrotic cells can not be labelled with b-dUTP, because either the number of DNA strand breaks is low or the 3′-OH termini in these breaks are not accessible to TdT, in contrast to cells undergoing apoptosis. Therefore, the fraction of cells undergoing necrosis cannot be determined using this method, because they cannot be separated from the normal cell population. In addition, the apoptotic cell fractions do not contain any necrotic cells. The determination of apoptotic cell fractions in HL-60 and HL-60/ara-C cells revealed that ara-C is able to induce this mechanism of cell death at much lower concentrations than NHAC, but only to a limited fraction of cells, which represents the S-phase cells, whereas the induction of apoptosis by NHAC is suggested not to be limited to S-phase cells (Tables I and III). This limitation of ara-C toxicity to S-phase cells, together with its rapid inactivation, makes it necessary to administer this drug *in vivo* either continuously for 5 days (Frei *et al.*, 1969) or at high-dose regimens up to 3 g $m^{-2}$ (Momparler, 1974). As demonstrated with incubations with high NHAC concentrations in both cell lines, the large number of cells undergoing apoptosis does not only depend on the formation of ara-CTP formed from NHAC. These small amounts of ara-C formed from NHAC are unlikely to induce apoptosis in HL-60/ara-C cells, as shown by the direct incubation with ara-C (Table III). NHAC itself (or one of its yet unknown metabolites, with the exception of ara-C) at concentrations ⩾ 200 μM is able to induce apoptosis even in HL-60/ara-C-resistant cells.

The comparison of the time-dependent studies of apoptosis induction with the cell cycle distribution clearly shows that the inhibition of DNA synthesis in HL-60 cells occurs within the first 2 h of incubation with ara-C, whereas apoptotic cells appear only after longer lasting drug exposure (Table IV). In HL-60/ara-C cells, NHAC also induced apoptosis 4–24 h after the beginning of incubation, however, at fractions not reaching more than 5.5% of the cell population. Ara-CTP formed from NHAC is unlikely to be responsible for the DNA fragmentation, as shown by direct incubation with ara-C.

In conclusion, our study gives further evidence that NHAC has significantly different cytotoxic mechanisms than ara-C and that this lipophilic derivative is able to overcome ara-C resistance. Thus, NHAC exerts at concentrations of 1–40 μM a phase-independent cytotoxicity and is able to induce apoptosis at higher concentrations, which is not restricted to S-phase cells. Together with its highly improved stability against deamination, these properties might explain why NHAC can be administered at lower doses and with single-dose schedules to obtain even better anti-tumour effects than ara-C in the murine L1210 leukaemia model (Schwendener and Schott, 1992).

The exact mechanism of action of NHAC, however, is not yet elucidated and further studies are required. One possible mechanism of action for NHAC, fitting into the findings described here, may consist in an interference of NHAC with signal transduction pathways, which was also described as a mechanism for synthetic anti-tumour alkyl ether lipids (Berggren *et al.*, 1993; Lohmeyer and Workman, 1993; Lohmeyer and Bittman, 1994; Houlihan *et al.*, 1995). Therefore, further investigations with NHAC will be necessary to clarify its effects on intracellular cell signalling. The chemical structure of NHAC with its highly lipophilic alkyl moiety and the ara-C component may also raise the assumption that its cytotoxic action consists in a combination of mechanisms such as those of the ether lipids and, at a reduced rate, those of ara-C.

**Acknowledgements**

The authors wish to thank E. Niederer for the flow cytometry measurements. This work was supported by the Stiftung für Krebsbekämpfung, the Stiftung für angewandte Krebsforschung and the Foundation for Medical Research of the University of Zurich.

### References

ARENDS MJ, MORRIS RG AND WYLLIE AH. (1990). Apoptosis. The role of the endonuclease. *Am. J. Pathol.*, **136**, 593–608.

BERGGREN MI, GALLEGOS A, DRESSLER LA, MODEST EJ AND POWIS G. (1993). Inhibition of the signaling enzyme phosphatidylinositol-3-kinase by antitumor ether lipid analogues. *Cancer Res.*, **53**, 4297–4302.

BERTRAND R, SARANG M, JENKIN J, KERRIGAN D AND POMMIER Y. (1991). Differential induction of secondary DNA fragmentation by topoisomerase II inhibitors in human tumor cell lines with amplified c-myc expression. *Cancer Res.*, **51**, 6280–6285.

BHALLA K, NAYAK R AND GRANT S. (1984). Isolation and characterization of a deoxycytidine kinase-deficient human promyelocytic leukemic cell line highly resistant to 1-β-D-arabinofuranosylcytosine. *Cancer Res.*, **44**, 5029–5037.

CAVAZZANA M, CALVO F, FACCHIN P, BARREAU P, GENY B, DAL CL AND DRESCH C. (1988). Use of ligand culture and cell cycle analysis to compare drug damage following *in vitro* treatment of normal human bone marrow cells with adriamycin, arabinosylcytosine, and etoposide. *Exp. Hematol.*, **16**, 876–883.

COHEN GM, SUN XM, SNOWDEN RT, DINSDALE D AND SKILLETER DN. (1992). Key morphological features of apoptosis may occur in the absence of internucleosomal DNA fragmentation. *Biochem. J.*, **286**, 331–334.

COHEN JJ. (1993). Apoptosis. *Immunol. Today*, **14**, 126–130.

FIETKAU R, FRIEDE H AND MAURER-SCHULTZE B. (1984). Cell kinetic studies of the cytostatic and cytocidal effect of 1-β-D-arabinofuranosylcytosine on the L1210 ascites tumor. *Cancer Res.*, **44**, 1105–1113.

FREI E, BICKERS JN, HEWLETT JS, LANE M, LEARY WV AND TALLEY RW. (1969). Dose schedule and antitumor studies of arabinosyl cytosine. *Cancer Res.*, **29**, 1325–1332.

GORCZYCA W, GONG J, ARDELT B, TRAGANOS F AND DARZYNKIEWICZ Z. (1993*a*). The cell cycle related differences in susceptibility of HL-60 cells to apoptosis induced by various antitumor agents. *Cancer Res.*, **53**, 3186–3192.

GORCZYCA W, BIGMAN K, MITTELMAN A, AHMED T, GONG J, MELAMED MR AND DARZYNKIEWICZ Z. (1993*b*). Induction of DNA strand breaks associated with apoptosis during treatment of leukemias. *Leukemia*, **7**, 659–670.

HOPE MJ, BALLY MB, WEBB G AND CULLIS PR. (1985). Production of large unilamellar vesicles by a rapid procedure, charactization of size distribution, trapped volume and ability to maintain a membrane potential. *Biochim. Biophys. Acta.*, **812**, 55–65.

HORBER DH, SCHOTT H AND SCHWENDENER RA. (1995*a*). Cellular pharmacology of a liposomal preparation of $N^4$-hexadecyl-1-β-D-arabinofuranosylcytosine, a lipophilic derivative of 1-β-D-arabinofuranosylcytosine. *Br. J. Cancer*, **71**, 957–962.

HORBER DH, SCHOTT H AND SCHWENDENER RA. (1995*b*). Cellular pharmacology of $N^4$-hexadecyl-1-β-D-arabinofuranosylcytosine (NHAC) in the human leukemic cell lines K-562 and U-937. *Cancer Chemother. Pharmacol.* (in press).

HORBER DH, OTTIGER C, SCHOTT H AND SCHWENDENER RA. (1995*c*). Pharmacokinetic properties and interactions with blood components of $N^4$-hexadecyl-1-β-D-arabinofuranosylcytosine (NHAC) incorporated into liposomes. *J. Pharm. Pharmacol.*, **47**, 282–288.

HOULIHAN WJ, LOHMEYER M, WORKMAN P AND CHEON SH. (1995). Phospholipid antitumor agents. *Med. Res. Rev.* (in press).

HOTZ MA, GONG J, TRAGANOS F AND DARZYNKIEWICZ Z. (1994). Flow cytometric detection of apoptosis: comparison of the assays of in situ DNA degradation and chromatin changes. *Cytometry*, **15**, 237–244.

KARON M AND SHIRAKAWA S. (1969). The locus of action of 1-β-D-arabinofuranosylcytosine in the cell cycle. *Cancer Res.*, **29**, 687–696.

KAUFMANN SH. (1989). Induction of endonucleolytic DNA cleavage in human acute myelogenous leukemia cells by etoposide, camptothecin, and other cytotoxic anticancer drugs: a cautionary note. *Cancer Res.*, **49**, 5870–5878.

KOLLA SS AND STUDZINSKI GP. (1994). Constitutive DNA binding of the low mobility forms of the AP-1 and SP-1 transcription factors in HL60 cells resistant to 1-β-D-arabinofuranosylcytosine. *Cancer Res.*, **54**, 1418–1421.

LACOMBE F, BELLOC F, BERNARD P AND BOISSEAU MR. (1988). Evaluation of four methods of DNA distribution data analysis based on bromodeoxyuridine/DNA bivariate data. *Cytometry*, **9**, 245–253.

LOHMEYER M AND BITTMAN R. (1994). Antitumor ether lipids and alkylphosphocholines. *Drugs Future*, **19**, 1021–1037.

LOHMEYER M AND WORKMAN P. (1993). The role of intracellular free calcium mobilization in the mechanism of action of antitumour ether lipids SRI 62-834 and ET18-OMe. *Biochem. Pharmacol.*, **45**, 77–86.

MOMPARLER RL. (1974). A model for the chemotherapy of acute leukemia with 1-β-D-arabinofuranosylcytosine. *Cancer Res.*, **34**, 1775–1787.

PREISLER HD, GOPAL V, BANAVALI SD, FINKE D AND BOKARI SA. (1992). Multiparameter assessment of the cell cycle effects of bioactive and cytotoxic agents. *Cancer Res.*, **52**, 4090–4095.

SCHWENDENER RA AND SCHOTT H. (1992). Treatment of L1210 murine leukemia with liposome-incorporated $N^4$-hexadecyl-1-β-D-arabinofuranosyl cytosine. *Int. J. Cancer*, **51**, 466–469.

SCHWENDENER RA, HORBER DH, OTTIGER C AND SCHOTT H. (1995). Preclinical properties of $N^4$-hexadecyl- and $N^4$-octadecyl-1-β-D-arabinofuranosylcytosine in liposomal preparations. *J. Liposome Res.*, **5**, 27–47.

WYLLIE AH, KERR JF AND CURRIE AR. (1980). Cell death: the significance of apoptosis. *Int. Rev. Cytol.*, **68**, 251–306.

YOSHIDA A, UEDA T, WANO Y AND NAKAMURA T. (1993). DNA damage and cell killing by camptothecin and its derivative in human leukemia HL-60 cells. *Jpn. J. Cancer Res.*, **84**, 566–573.